# Beta-Adrenoceptor Activation Reduces Both Dermal Microvascular Endothelial Cell Migration via a cAMP-Dependent Mechanism and Wound Angiogenesis

**DOI:** 10.1002/jcp.24716

**Published:** 2014-10-28

**Authors:** Andrew P O'Leary, James M Fox, Christine E Pullar

**Affiliations:** Department of Cell Physiology and Pharmacology, University of LeicesterLeicester, UK

## Abstract

Angiogenesis is an essential process during tissue regeneration; however, the amount of angiogenesis directly correlates with the level of wound scarring. Angiogenesis is lower in scar-free foetal wounds while angiogenesis is raised and abnormal in pathophysiological scarring such as hypertrophic scars and keloids. Delineating the mechanisms that modulate angiogenesis and could reduce scarring would be clinically useful. Beta-adrenoceptors (β-AR) are G protein-coupled receptors (GPCRs) expressed on all skin cell-types. They play a role in wound repair but their specific role in angiogenesis is unknown. In this study, a range of in vitro assays (single cell migration, scratch wound healing, ELISAs for angiogenic growth factors and tubule formation) were performed with human dermal microvascular endothelial cells (HDMEC) to investigate and dissect mechanisms underpinning β-AR-mediated modulation of angiogenesis in chick chorioallantoic membranes (CAM) and murine excisional skin wounds. β-AR activation reduced HDMEC migration via cyclic adenosine monophosphate (cAMP)-dependent and protein kinase A (PKA)-independent mechanisms as demonstrated through use of an EPAC agonist that auto-inhibited the cAMP-mediated β-AR transduced reduction in HDMEC motility; a PKA inhibitor was, conversely, ineffective. ELISA studies demonstrated that β-AR activation reduced pro-angiogenic growth factor secretion from HDMECs (fibroblast growth factor 2) and keratinocytes (vascular endothelial growth factor A) revealing possible β-AR-mediated autocrine and paracrine anti-angiogenic mechanisms. In more complex environments, β-AR activation delayed HDMEC tubule formation and decreased angiogenesis both in the CAM assay and in murine excisional skin wounds in vivo. β-AR activation reduced HDMEC function in vitro and angiogenesis in vivo; therefore, β-AR agonists could be promising anti-angiogenic modulators in skin. J. Cell. Physiol. 230: 356–365, 2015. © 2014 The Authors. *Journal of Cellular Physiology* Published by Wiley Periodicals, Inc.

Angiogenesis is the formation of new blood vessels from pre-existing post capillary venules; it plays an important role in development, tissue regeneration and tumour growth. Endothelial cells (EC) differentiate and detach from adjacent capillaries, proliferate and migrate directionally. ECs then align and elongate to form leaky tubules that connect to form loops. The newly formed tubules mature and are reinforced by the recruitment of periendothelial support cells (Bauer et al., 2005; Eming et al., 2007). Angiogenesis is essential for wound repair (Falanga, 2005) and the amount of wound angiogenesis directly correlates with the level of scarring (van der Veer et al., 2011). Vascular endothelial growth factor (VEGF)-A addition to wounds promotes scarring and levels of VEGF and angiogenesis are lower in scar-free fetal wounds (Wilgus et al., 2008) and non-scarring oral wounds (Szpaderska et al., 2005). Excessive angiogenesis occurs, and persists, in hypertrophic scars from 12 weeks postoperatively (van der Veer et al., 2011) and in keloid scars (Mogili et al., 2012). The mechanisms by which Beta-adrenoceptors (β-AR) modulate dermal EC function in the angiogenic process during skin wound healing have largely been unexplored.

β-ARs are G protein-coupled receptors (GPCRs) for the endogenous catecholamines, adrenaline and noradrenaline (Hall, 2004). There are three β-AR subtypes: β_1_-AR, β_2_-AR, and β_3_-AR, which differ in their protein sequences and respond differently to their catecholamine ligands (Hall, 2004). β_1_-AR, β_2_-AR (Ponicke et al., 2006) and β_3_-AR (Soeder et al., 1999) can all couple to Gαs activating the membrane effector enzyme adenylate cyclase (AC) which generates the secondary messenger molecule cyclic adenosine monophosphate (cAMP) by catalysing the conversion of adenosine triphosphate to cAMP (Gilman, 1987; Hamm, 1998). Intracellular changes in cAMP levels can alter a multitude of cell functions including rat bone marrow progenitor EC migration (Chen et al., 2010), rat aortic EC proliferation (Torella et al., 2009) and chick chorioallantoic membranes (CAM) angiogenesis (Pacini et al., 2011) through the downstream action of cAMP-dependent protein kinase A (PKA) and exchange protein directly activated by cAMP (EPAC) (de Rooij et al., 1998). Prior to 1998, any intracellular cAMP effects were attributed solely to PKA; the discovery that the small GTPase Rap1 can be directly activated by EPAC, a family of cAMP-activated guanine nucleotide exchange factors (GEFs), heralded a new era in cAMP research (Kawasaki et al., 1998; de Rooij et al., 1998). EPAC1 and EPAC2 are multidomain proteins containing an autoinhibitory cAMP-binding domain that inhibits EPAC enzymatic activity in the absence of cAMP. cAMP relieves this auto-inhibition. EPAC1 and EPAC2 also contain dishevelled, Egl-10 and pleckstrin homology domains that play a role in membrane localisation (de Rooij et al., 2000). EPAC1 is broadly expressed while EPAC2 expression is restricted to the brain and adrenal glands (Kawasaki et al., 1998; de Rooij et al., 1998).

Previous genetic and functional research has implicated the β-AR family in the regulation of angiogenesis. In a genetic study, investigating choroidal neovascularisation that used composite interval mapping to identify two new quantitative trait loci on chromosomes 2 and 19, several pro-angiogenic genes were identified, including the β_2_-AR gene (Nakai et al., 2009). Functionally, β_2_-AR stimulation can induce nitric oxide release (Ferro et al., 1999) altering vessel tone and transendothelial permeability (Baluk and McDonald, 1994; Zink et al., 1995). Selective β_2_-AR antagonist treatment increased angiogenesis in the aortic outgrowth assay, the CAM assay and in an excisional mouse wound model in vivo (Pullar et al., 2012). Correspondingly, blockade of the β-ARs, with the non-selective antagonist propranolol, increased angiogenesis in a rat cutaneous wound-healing model (Romana-Souza et al., 2006, 2009; Romana-Souza and Monte-Alto-Costa, 2010; Romana-Souza et al., 2010).

Here, we investigate the effect of isoproterenol (Iso), a non-selective β-AR full agonist, exhibiting broad selectivity for β_1_-AR, β_2_-AR and β_3_-AR (Baker, 2010), on human dermal microvascular endothelial cell (HDMEC) migration, growth factor secretion and tubule formation, functions they perform during the process of angiogenesis in skin wound healing. We explored the effects of Iso on angiogenesis in both the developing chick embryo and in regenerating murine skin wounds and probed the mechanisms underpinning its actions. This work will provide a novel insight into the modulation of angiogenesis in healing skin, which may have clinical relevance in reducing normal wound scarring and the excessive scarring observed in hypertrophic scars and keloids.

## Materials and Methods

### Reagents

Tissue culture reagents were from Invitrogen (Paisley, UK), unless otherwise stated. Iso hydrochloride and all other pharmacological reagents were from Tocris Bioscience (Bristol, UK) or Calbiochem (Nottingham, UK).

### Cell culture

All cells were grown at 37 °C with 5% CO_2_/95% air in a humidified atmosphere. Primary HDMECs, isolated from human neonatal foreskin dermis, were purchased from Invitrogen. At least three separate strains, isolated from human neonatal dermis from different males, were used between passages 3 and 7, to obtain consistent data in all in vitro experiments. Above passage 7, cells become senescent and morphological changes occur, such as increase in cell size. Cells were grown in endothelial cell growth medium (ECGM) containing microvascular growth supplement (MGS) (5% foetal bovine serum, 0.4% endothelial cell growth supplement, 10 ng/ml epidermal growth factor (GF), 10 ng/ml fibroblast growth factor (FGF)-2, 20 ng/ml insulin-like growth factor, 0.5 ng/ml VEGF 165, 1 μg/ml ascorbic acid, 90 µg/ml heparin and 1 μg/ml hydrocortisone) (PromoCell, Heidelberg, Germany), 100 U/ml penicillin and 100 μg/ml streptomycin.

Primary human neonatal keratinocytes (HNKs), isolated from human neonatal foreskin epidermis, were purchased from Invitrogen. At least three separate strains from different male neonatal epidermises were used between passages 3 and 7 and were grown in keratinocyte growth medium (KGM) with 60 μM Ca^2+^ supplemented with human growth supplement (0.2 ng/ml epidermal growth factor, 1 μg/ml human-insulin-like growth factor-1, 5 μg/ml bovine transferrin, 0.18 μg/ml hydrocortisone and 0.2% bovine pituitary extract), 100 U/ml penicillin and 100 μg/ml streptomycin.

### Western blotting

2 × 10^5^ HDMECs or HNKs were seeded in a 90 mm culture dish and incubated for 24 h. Cells were placed on ice and washed twice with 3 ml ice-cold PBS containing phosphatase inhibitors (50 mM NaF and 1 mM Na_3_VO_4_) then scraped in lysis buffer (PBS containing 50 mM NaF, 1 mM Na_3_VO_4_, 0.5% Triton X-100, 200 μg/ml PMSF, 1X protease inhibitor cocktail) (Roche, Welwyn Garden City, UK). The lysates were centrifuged at 14,000*g* for 10 min at 4 °C. The protein concentration of each sample was determined using the Bradford protein assay (Biorad, Hemel Hempstead, UK).

HDMEC lysates were added to sample buffer containing dithiothreitol. Samples (55 μg/lane (HDMEC); 83 μg/lane (HNK)) were electrophoretically separated on 10% Bis-tris gels (Biorad) and proteins were transferred to polyvinylidene fluoride membranes (Roche). The membranes were immunoblotted with 1 μg/ml anti-β_1_-AR (77189, goat polyclonal), 0.5 μg/ml anti-β_2_-AR (71219, rabbit polyclonal), 0.1 μg/ml anti-β_3_-AR (77588, goat polyclonal) and 0.1 μg/ml β-actin (8229 (goat); 8227 (rabbit)) as a loading control (Abcam, Cambridge, UK). Blots were developed using electro-chemiluminescence detection agents (GE Healthcare, Amersham, UK).

### Single cell migration assay

HDMECs from three different donors were plated at 3.5 × 10^3^ cells/cm^2^ on collagen I-coated (30 μg/ml; Cohesion Technologies, Invitrogen) 35 mm plastic cell culture dishes for 2 h at 37 °C. In some experiments, HDMECs were serum-starved for 24 h in ECGM without MGS. HDMECs were incubated with ECGM alone or ECGM containing Iso at time 0 or following a 60 min preincubation. The 35 mm dishes were placed in a heating chamber at 37 °C on a Nikon Eclipse phase contrast microscope. Time-lapse images were taken every 10 min over a 60 min period using a Hamamatsu digital camera under automation via Volocity or Openlab software (v5.0 or v5.5.2, Perkin Elmer, Coventry, UK) and cell tracking was performed with the same software (Pullar et al., 2007). A control experiment was always performed to ensure that the cells were healthy. Cells (15–40) were imaged and tracked from control or treatment experiments with the cell data for each group being pooled from a minimum of four individual experiments prior to analysis. The cell data from all control experiments (88) were pooled to give a large total cell number (2219). The cell data from all experiments for a particular treatment were pooled to give a minimum pooled cell number above 60 (a minimum of 15 cells from each of four experiments). The experiments performed in Figure 2 and Figure 4 were all performed under the same control conditions (no pre-treatments; ECGM alone or treatments added at time 0) and therefore the same pooled control data set was used.

### Scratch assay

HDMECs were seeded at 3 × 10^4^ cells/well in triplicate on collagen I-coated (30 μg/ml) 48-well plates. A pipette tip was used to scratch a 1 mm-wide gap in the well centre, creating two wound edges. Wells were then HBSS washed and media was added either alone or containing 10 µM Iso. The demarcated areas of each well were then photographed at time 0, 6, 12, 24 and 32 h under a Nikon Eclipse phase contrast microscope using a Hamamatsu digital camera and Improvision software (Perkin Elmer), as previously described (Pullar et al., 2006). The data were analysed using NIH Image 1.6 (http://rsb.info.nih.gov/nih-image) to determine the remaining uncovered area.

### Human FGF-2 and VEGF-A ELISAs

HDMECs and HNKs were grown to approximately 75% confluence before seeding at 80 × 10^4^ cells/well in 6-well plates. Cells were washed with HBSS and then serum starved for 24 h. HDMECs were incubated in ECGM containing 2% FBS in the absence or presence of 10 μM Iso for 6, 24 and 48 h. HNKs were also incubated for 6, 24 and 48 h with either KGM alone or KGM containing 10 μM Iso. Human FGF-2 and VEGF-A levels were determined following the manufacturers' instructions for Quantikine Duoset ELISA kits (R&D Systems, Abingdon, UK) using a TECAN Nanoquant plate reader (Tecan UK Ltd. Reading, UK).

### Tubule formation assay

Cultrex basement membrane extract (BME) (AMS Biotech, Abingdon, UK) was thawed on ice at 4 °C before 50 μl was added to the centre of required wells on a 96-well plate and incubated for 30 min at 37 °C to facilitate gelation. HDMECs were grown to approximately 75% confluence, trypsinised and 50 μl containing 20 × 10^4^ cells, was added on top of the BME before a 2 h incubation at 37 °C. A 50 μl of either media alone or media containing 20 μM Iso was added to each well and the wells were photographed using a Hamamatsu digital camera on a Nikon eclipse phase contrast microscope at 0 and 6 h using Openlab software (Improvision, Perkin Elmer). Tubule formation was analysed by counting the number of tubule-like structures in five images, taken at pre-determined positions in each well, in duplicate.

### Animals and ethics

FVB/N mice were purchased from The Jackson Laboratory: females aged between 8 and 12 weeks were used in the study. The University of California Davis Institutional Animal Care and Use Committee approved the animal protocol (number 04–11523). All murine wound surgeries were performed under ketamine/xylazine anaesthesia and all efforts were made to minimise suffering.

Under the UK Animals (Scientific Procedures) Act 1986, a vertebrate embryo only becomes a “protected animal” when two-thirds of the gestation period (21 days) has elapsed. As the fertilised hen eggs, used in the CAM assay, were disposed of after 10 days no Institutional or Government approval was required.

### Chick chorioallantoic membrane (CAM) assay

Fertilised eggs were obtained through the Biomedical Sciences Division, University of Leicester, from local hatcheries and incubated at 37 °C in a humidified environment for 48 h. On day 3, approximately 5 ml of albumin was removed from the obtuse poles of the eggs using a 21 G cannula needle. Square windows (2 × 2 cm^2^) were created by drilling through the shell and shell membrane of the egg using a Dremel cutter (Dremel, Uxbridge, UK). The windows were sealed with parafilm and tape and the eggs were incubated in a horizontal position at 37 °C in a humidified environment for a further 48 h. On day 5, 10 μM Iso or 50 μM sp cAMP treatments were dried onto the centre of sterile 13 mm circular, 0.16 mm thick, glass coverslips (VWR, Lutterworth, UK) for 2 h and then placed face down onto the CAMs, accessible through the window. CAMs were photographed at the centre of each coverslip every 24 h until day 10 using a prior stereomicroscope and a premiere digital microscope eyepiece (Model MA88) (Prior Scientific, Cambridge, UK). Images were analysed by counting the total number of vessel branch points per field of view. The average total number of vessel branch points per field of view was then calculated for control and treatment groups as an indicator for the amount of angiogenesis. All fertilised eggs were disposed of 10 days post fertilisation.

### Murine wound model

Mice housed separately were anaesthetised by intraperitoneal injection of ketamine (100 mg/kg)/xylazine (10 mg/kg) (Pfizer, Sandwich, UK). Back skin was shaved and two circular, full-thickness, 6 mm excisional wounds were created with scissors, 2 cm apart, in the centre of the back, using a sterile biopsy punch (SMS Inc., Columbia, MD, USA) and marked with permanent ink dots. Wounds were treated topically with 100 μl of hydrogel alone (Duoderm, ConvaTec, Flintshire, UK) or containing 0.1% salbutamol (approximately 1.7 mM) which should activate all β-ARs (Baker, 2010), a dose previously used to topically suppress allergic reactions (Gautheron and Sugrue, 1987), immediately after wounding and daily until harvesting. Wounds were harvested at 5 days post-wounding by carefully applying an 8 mm punch (SMS Inc.) around the original wound site then excising the wound and surrounding tissue using scissors.

For histological analysis, biopsies were fixed in IHC zinc fixative (BD Biosciences, Oxford, UK), bisected to ensure sections were from the wound centre then dehydrated through an ethanol-xylene series and embedded in paraffin. A 7 μm-thick cryo-sections were stained with anti-mouse CD31 (3.125 μg/ml, BD Biosciences) and revealed using an anti-Ig HRP detection kit (BD Biosciences), according to manufacturer's protocols. Specimens that were damaged in the histologic process or otherwise non-interpretable were excluded from the study. Numbers of stained vessels in ten fields of view, from the stained section from each mouse, were counted in a double-blind manner and the average vessel number was calculated for each group (N = 10–14).

### Statistical analysis

The data were averaged and statistically analysed in GraphPad Prism v6.02 (GraphPad Software, Inc. La Jolla, CA, USA) using a non-parametric *t*-test to compare two parameters or one-way ANOVA followed by the Dunnett's test for multiple parameter analysis. The values were graphically represented with the bars representing the means ± SEM. Significance was ascribed as * for *P* < 0.05 and ** for *P* < 0.01.

## Results

### HDMECs express all three subtypes of β-AR

**Fig 1:**
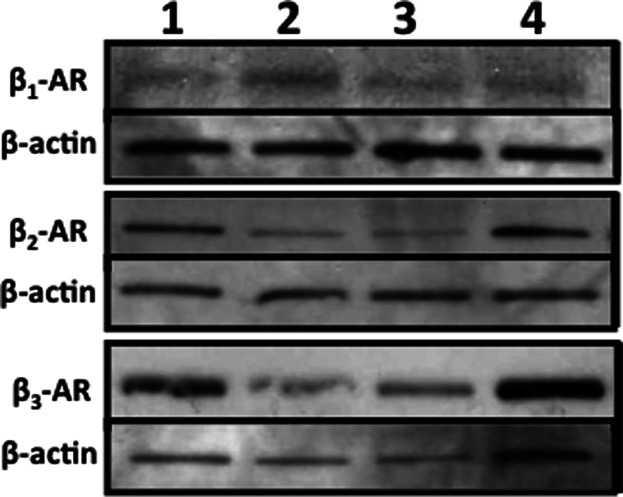
All three β-ARs are detected in HDMECs. Four different HDMEC strains (1–4) were lysed, proteins were separated electrophoretically and membranes were immunoblotted with antibodies specific for β_1_-AR (51 kDa), β_2_-AR (47 kDa), β_3_-AR (55 kDa) and β-actin (40 kDa) before chemiluminescent detection, as described in the methods.

β-AR expression differs between EC type: adult human iliac vein, bovine fetal aortic (Howell et al., 1988), bovine aortic and bovine pulmonary aortic (Ahmad et al., 1990) ECs express both β_1_-AR and β_2_-AR. Retinal ECs express both β_1_-AR and β_3_-AR (Steinle, 2003), while choroidal ECs express β_1_-AR, β_2_-AR and β_3_-AR (Steinberg et al., 1984; Howell et al., 1988; Steinle et al., 2003; Steinle et al., 2005). However, until now, the composition of β-AR expression in primary HDMECs was not known. Western blotting studies revealed that HDMECs express the full repertoire of β-AR subtypes: β_1_-AR, β_2_-AR and β_3_-AR (Fig. 1).

### β-AR activation decreased HDMEC single cell migration rate

**Fig 2:**
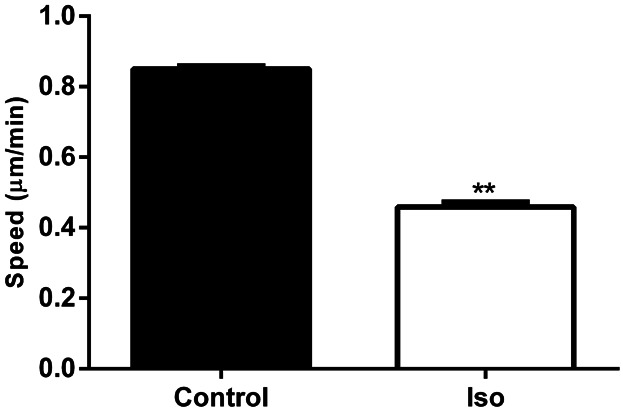
β-AR activation decreases the rate of HDMEC single cell migration. Time lapse images of untreated HDMECs (3 different strains) or those treated with 10 μM Iso were taken over a 60 min period allowing the average rate of migration of single HDMECs to be calculated via cell tracking using Openlab software (N = number of individual cells from 10 (Iso) and 88 (control) independent experiments; control N = 2219; Iso 10 μM N = 382). Individual cell data were averaged, statistically analysed and graphically represented with the bars representing the means ± SEM. (**P < 0.01).

EC migration is an important early step in skin wound angiogenesis (Lamalice et al., 2007). To investigate this, HDMEC single cell migration (SCM) was performed. HDMEC migrated randomly at a rate of 0.85 + 0.008 μm/min. The addition of β-AR agonist (Iso; 10 μM) significantly reduced HDMEC SCM rate by 46% (Fig. 2). Lower concentrations of Iso reduced HDMEC migration, but not significantly (results not shown), therefore a concentration of 10 μM was chosen for further mechanistic studies. To determine if pre-incubation with β-AR agonist would have an additional effect on HDMEC SCM, HDMECs were pre-treated for 60 min prior to the start of the one-hour experiments. However, pre-incubating with β-AR agonist had no further effect (data not shown). Moreover, serum in the media could influence HDMEC SCM; therefore, HDMECs were serum-starved for 24 h prior to SCM. Indeed, in the absence of serum, spontaneous HDMEC migration rate was reduced by 43%. Nevertheless, β-AR agonist maintained its ability to reduce HDMEC migration speed, significantly reducing migration rate by 36% (data not shown).

Previous mechanistic studies have revealed that human keratinocytes could synthesise and secrete adrenaline (Schallreuter et al., 1992; Schallreuter, 1997; Pullar et al., 2006), which inhibited keratinocyte migration. To investigate if a similar mechanism existed in HDMECs, migration experiments were performed in the presence of a non-selective β-AR antagonist; however, it had no effect on cell migration rate (results not shown). In addition, HDMECs did not express tyrosine hydroxylase; a critical catecholamine synthesis enzyme (Nagatsu et al., 1964) and we could not detect adrenaline secretion from HDMECs by enzyme immune assay (results not shown).

### β-AR activation delayed scratch wound closure

**Fig 3:**
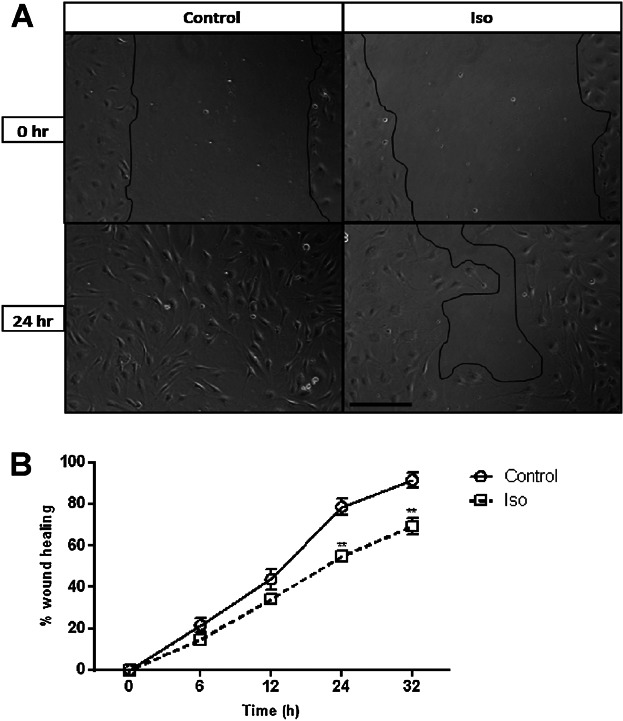
β-AR activation delays HDMEC scratch wound closure. Scratch wound assays were performed as described in the methods. Cells were treated with media alone or media containing 10 µM Iso. Demarcated areas of each well were photographed 0, 6, 12, 24 and 32 h later. Images representative of control and Iso-treated wounds at time 0 and 24 h are presented; scale bar = 200 μm (A). Combined data from 5–6 independent experiments using 3 s separate cell strains is shown after averaging, statistical analysis and graphical representation with the bars representing the means ± SEM (control N = 6; Iso N = 5) (**P < 0.01) (B).

To investigate wound angiogenesis in vitro, the scratch wound assay was used to determine the effect of Iso on HDMEC migration from a monolayer wound edge. β-AR activation delayed wound closure at 24 h (Fig. 3A/B). After 32 h, control wounds were 92% healed, while closure of β-AR agonist-treated wounds were significantly delayed by 25% (Fig. 3B).

### cAMP-dependent but PKA-independent pathways transduced the β-AR-mediated decrease in HDMEC single cell migration, while an EPAC modulator prevented the β-AR-mediated reduction in migration

**Fig 4:**
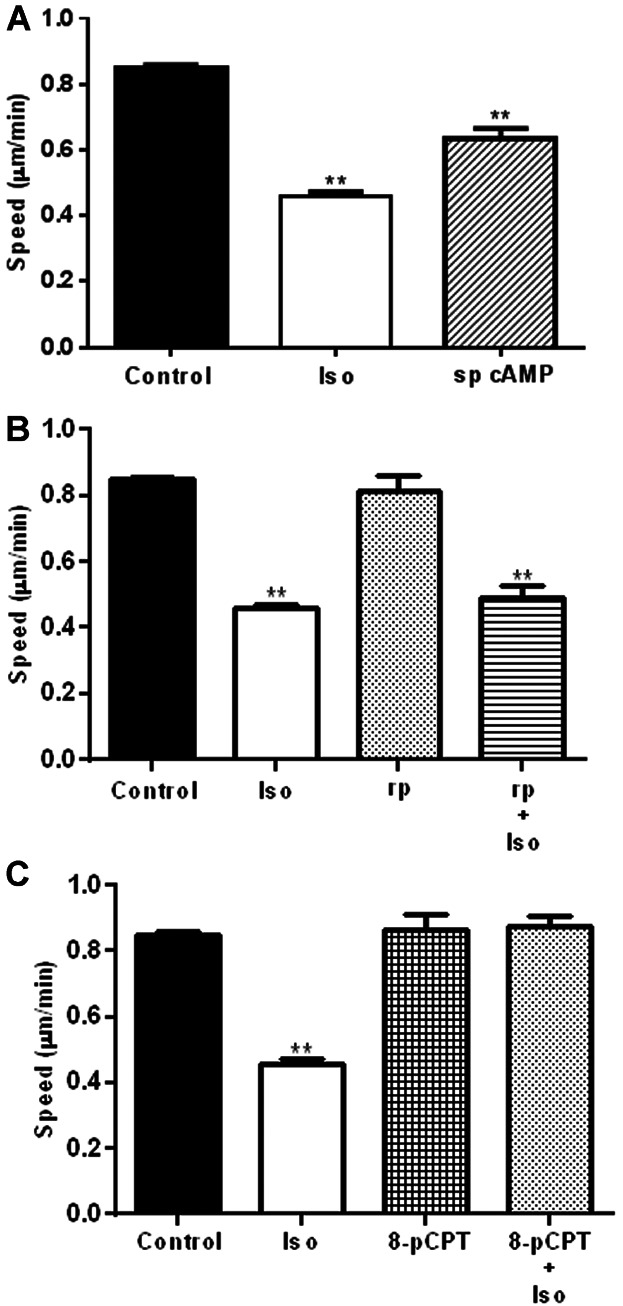
cAMP and EPAC-dependent pathways transduce the β-AR-mediated decrease in HDMEC single cell migration. The rate of HDMEC cell migration was determined as described in methods; the rate of migration of an average of N untreated cells or those treated with 10 μM Iso are shown for comparison on each panel and alongside cells treated with 50 μM sp cAMP (sp) (control N = 2219; Iso N = 382; sp cAMP N = 132) (A). HDMECs pre-incubated with the PKA inhibitor rp cAMP (rp; 50 µM) for 1 h before imaging and the addition of either 50 μM rp alone or in combination with 10 μM Iso (control N = 2219; Iso N = 382; rp cAMP N = 70; rp cAMP + Iso N = 81) (B). HDMECs pre-incubated with the EPAC agonist 8-pCPT (10 μM) for 1 h before the addition of 10 μM 8-pCPT alone or in combination with 10 μM Iso (control N = 2219; Iso N = 382; 8-pCPT N = 105; 8-pCPT + Iso N = 103) (C). Data were combined from 3–88 independent experiments using three separate cell strains. The data were averaged, statistically analysed and graphically represented with the bars representing the means ± SEM. (**P < 0.01).

To determine if cAMP played a role in the Iso mediated reduction in HDMEC migration rate, an active cAMP analogue sp cAMP, was used to increase intracellular cAMP levels. Sp cAMP decreased SCM by 25% (Fig. 4 A).

To investigate the role of cAMP-dependent protein kinases in the Iso mediated reduction in HDMEC motility, an inhibitor of PKA (rp cAMP (rp)) and an EPAC activator (8-CPT-2′-O-Me-cAMP (8-pCPT)) were used. 8-pCPT is a selective agonist for EPAC1 (Enserink et al., 2002) that activates EPAC1 with a higher affinity (EC_50_ 2.2 μM) than cAMP (EC_50_ 30 μM) but has no effect on PKA (Enserink et al., 2002).

The PKA inhibitor, rp, had no effect on cell migration alone and did not alter the Iso-mediated decrease in HDMEC migration rate (Fig. 4B). In contrast, while the EPAC agonist 8-pCPT had no effect on HDMEC migration alone, it completely prevented the Iso-mediated decrease in HDMEC SCM (Fig. 4C).

### β-AR activation decreased pro-angiogenic growth factor secretion

**Fig 5:**
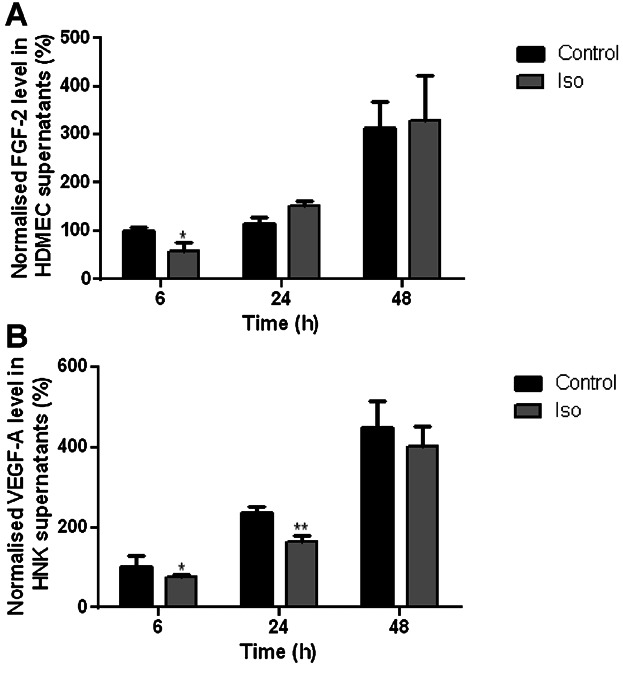
β-AR activation decreases FGF-2 and VEGF-A secretion from HDMECs and HNKs, respectively. Growth factor levels were determined using Duoset ELISA kits in supernatants from cells treated for 6, 24 and 48 h with basal media alone or containing 10 μM Iso for either human FGF-2 from HDMECs (A) or human VEGF-A from HNKs (B). Data were combined from four (HDMEC) or five (HNK) independent experiments using three separate cell strains. Data were averaged, statistically analysed and graphically represented with the bars representing the means ± SEM (*P < 0.05).

FGF-2 and VEGF are both pro-angiogenic growth factors that promote EC migration and proliferation (Nissen et al., 1998; Bouis et al., 2006). FGF-2 is synthesised and secreted by a number of wound cells such as dermal fibroblasts and ECs (Schweigerer et al., 1987; Kandel et al., 1991), while keratinocytes are a major source of VEGF in the wound (Brown et al., 1992). Keratinocytes express β_2_-ARs (Steinkraus et al., 1992). To determine if non-selective β-AR activation could alter growth factor secretion, FGF-2 and VEGF-A ELISA studies were performed on supernatants recovered from both HDMECs and HNKs after incubation in the presence or absence of Iso for 6, 24 and 48 h. HDMECs secreted FGF-2. The amount of FGF-2 secreted from control-treated HDMEC's was 12.4, 15.9 and 34.9 pg/ml after 6, 24 and 48 h, respectively. FGF-2 secretion was significantly reduced by Iso treatment after 6 h by 40%, while there was no significant difference in the amount of FGF-2 secreted between groups after 24 and 48 h (Fig. 5A). VEGF-A was secreted by HDMEC supernatants (< 10 pg/ml) but β-AR activation had no effect on its secretion (data not shown), while FGF-2 was secreted from HNKs (< 40 pg/ml), but β-AR activation also had no effect on its secretion (data not shown). The amount of VEGF-A secreted from control-treated HNK's was 155.3, 513.5 and 966.6 pg/ml after 6, 24 and 48 h, respectively. Iso significantly reduced HNK VEGF-A secretion by 25% after 6 h and by 37% after 24 h, while there was no significant difference in the amount of VEGF-A secreted between groups after 48 h (Fig. 5B).

### β-AR activation reduced HDMEC tubule formation

**Fig 6:**
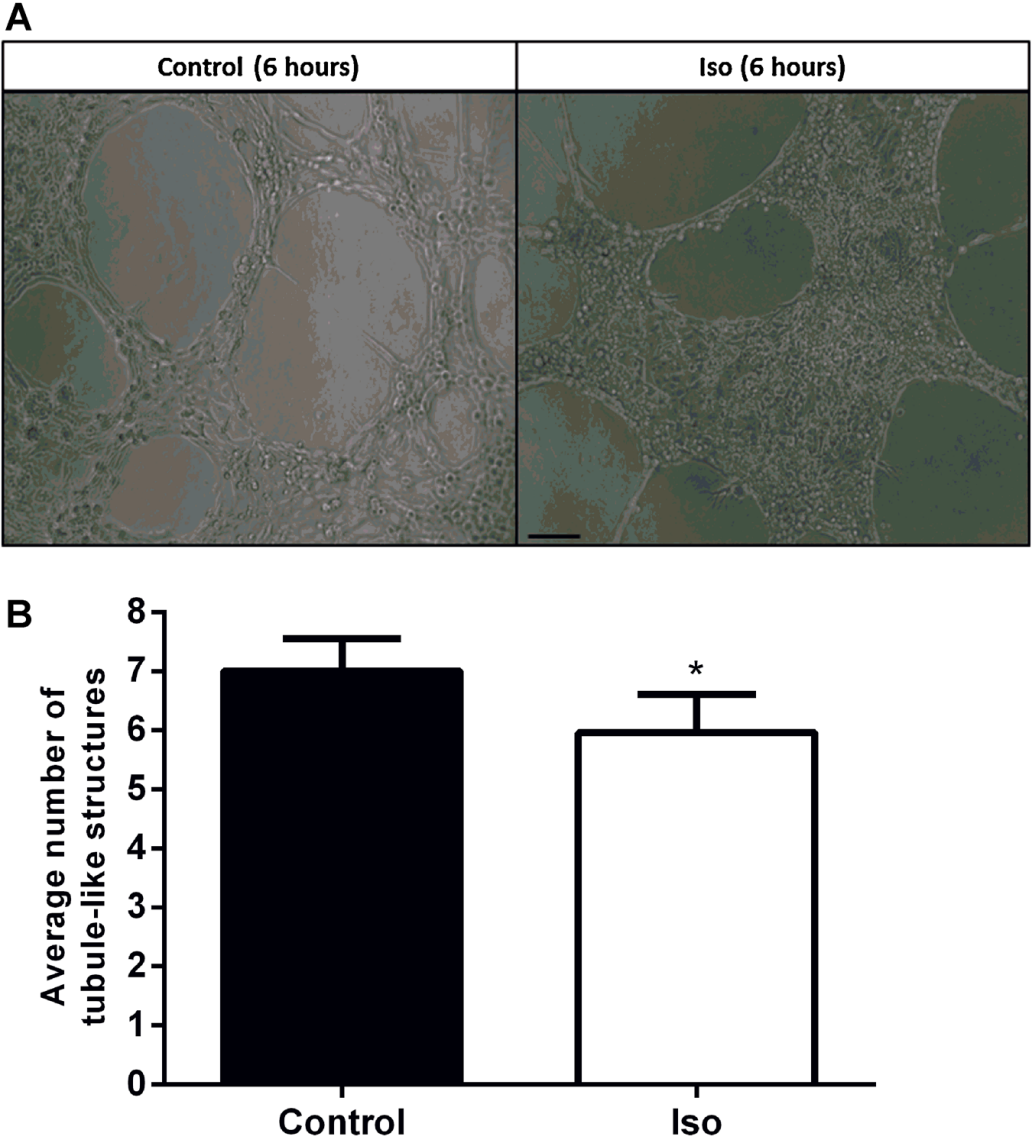
β-AR activation delays the formation of tubule-like structures in HDMECs. Tubule assays were performed as described in the methods. Untreated HDMECs or those treated with 10 μM Iso were imaged at 0 and 6 h. Images representative of control and Iso-treated cells at 6 h are presented; scale bar = 100 μm (A). Tubule formation was analysed by counting the number of tubule-like structures in five images, taken at pre-determined positions in each well, in duplicate. The data shown were combined from five independent experiments, from three separate cell strains. Data were averaged, statistically analysed and graphically represented with the bars representing the means ± SEM. (* *P* < 0.05) (B).

ECs undergo numerous physiological processes that contribute to angiogenesis, these include: invasion, alignment, elongation and apoptosis in addition to migration and proliferation (Bauer et al., 2005; Eming et al., 2007). To explore HDMEC physiological processes in a more complex environment, HDMECs were cultured on top of BME for 24 h in the presence or absence of Iso. HDMECs formed tubule-like structures after 6 h (Figs. 6A and B). To quantitate HDMEC tubule development, the number of tubule-like structures were counted. After 6 h, β-AR activation significantly delayed the formation of tubule-like structures by 14% (Fig. 6B).

### β-AR activation and an active cAMP analog reduced embryonic angiogenesis in the CAM assay

**Fig 7:**
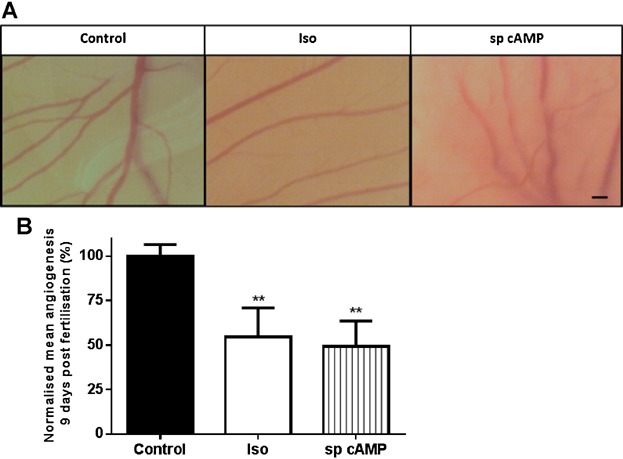
β-AR activation and sp cAMP decreases embryonic angiogenesis. CAM assays were performed as described. The CAMs were treated with 10 µM Iso or 50 µM sp cAMP at day 5. Eggs were imaged every 24 h until day 10. Images depicting representative angiogenesis 9 days post fertilisation are presented; scale bar = 1 mm (A). Images were analysed by counting the total number of vessel branch points per field of view. The mean total number of vessel branch points per field of view was then calculated for control and treatment groups to give a mean amount of angiogenesis. The data shown were combined from 3–16 independent experiments using a total of 28 eggs (control N = 16; Iso N = 9; sp cAMP N = 3). Data were statistically analysed, normalised to the control mean value for angiogenesis and graphically represented with the bars representing the means ± SEM. (** *P* < 0.01) (B).

To investigate the effect of β-AR activation on embryonic angiogenesis, the CAM assay was performed, as described (Ausprunk et al., 1974). Representative images of CAMs 9 days post-fertilisation are presented (Fig. 7A). Iso significantly decreased angiogenesis by 45%. Similarly, an active cAMP analogue, sp cAMP, significantly decreased angiogenesis by 51% (Fig. 7B).

### β-AR activation reduced angiogenesis in murine skin wounds in vivo

**Fig 8:**
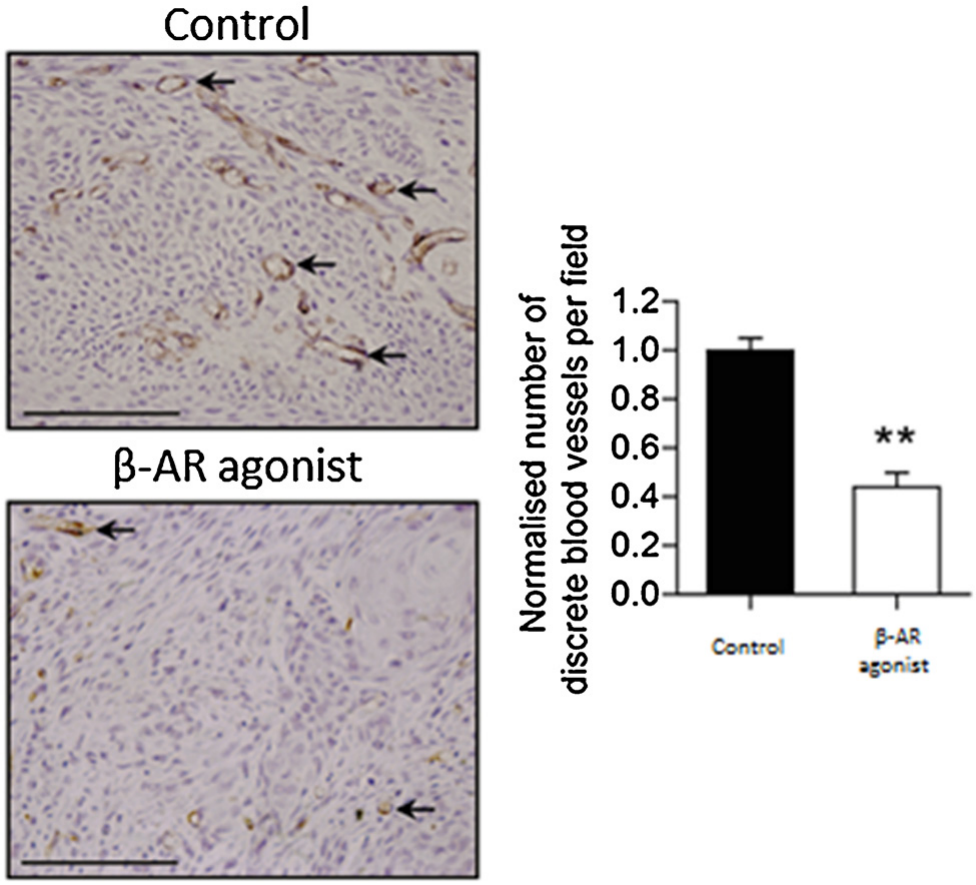
β-AR activation decreases murine skin wound angiogenesis in vivo. Angiogenesis was assessed by analysing CD31 immunostaining of 7 μm wound sections from control and 0.1% salbutamol-treated mice 5 days post-wounding. The number of stained vessels in 10 fields of view, from one stained section per mouse, were counted in a double-blind manner and the average vessel number was calculated for both groups (N = 10–14). Arrows point to examples of CD31-positive vessels (A). Data were averaged, statistically analysed and graphically represented with the bars representing the means ± SEM (** *P* < 0.01) (B).

To evaluate if β-AR activation altered skin wound angiogenesis, excisional murine skin wounds were treated daily with gel alone or containing 0.1% salbutamol (approximately 1.7 mM) which, at this concentration, similar to Iso, can activate all β-ARs (Baker, 2010). Salbutamol was chosen for the in vivo study as it is already widely used to treat asthma (Boskabady and Saadatinejad, 2003), therefore, should a potential clinical application be discovered, translation to the clinic could be fairly rapid through a repurposing of medicine route. Sections of murine wounds excised after 5 days were immunostained with an antibody to CD31. β-AR activation significantly reduced the number of blood vessels by an average of 56% (Fig. 8).

## Discussion

Our initial finding that all three β-AR subtypes are expressed by primary HDMECs (Fig. 1) further complements previous research exploring β-AR expression in ECs (Howell et al., 1988) and provided us with the stimulus to investigate the role that the β-ARs and their signalling networks might play in modulating the functional behaviour of this cell type. β-AR activation was found to be anti-motogenic, decreasing HDMEC SCM (Fig. 2) and scratch wound healing (Fig. 3). The β-AR mediated anti-motogenicity was transduced through cAMP-dependent, PKA-independent, pathways perhaps via EPAC (Fig. 4). ELISA studies demonstrated that β-AR activation reduced FGF-2 secretion from HDMECs and VEGF-A secretion from keratinocytes (Fig. 5). These autocrine and paracrine mechanisms may contribute to the β-AR mediated modulation of EC function in vitro and angiogenesis in vivo, respectively. Given the importance of EC migration to the initial stages of angiogenesis (Lamalice et al., 2007) it is, therefore, not surprising that in more complex environments we demonstrate that β-AR activation delayed tubule formation on BME (Fig. 6) and reduced angiogenesis both in the CAM assay (Fig. 7) and in murine excisional skin wounds in vivo (Fig. 8).

In human keratinocytes, β-AR activation also decreased cell migration rate (Pullar et al., 2003), while β-AR blockade increased motility (Pullar et al., 2006). Mechanistic studies have previously revealed that keratinocytes have the full capacity to synthesise and secrete the catecholamine adrenaline, generating an autocrine adrenergic network in the epidermis (Schallreuter et al., 1992; Schallreuter, 1997; Pullar et al., 2006), capable of retarding keratinocyte migration (Pullar et al., 2006). Investigations herein demonstrated that a similar mechanism did not exist in HDMECs, as a non-selective β-AR antagonist had no effect on cell migration rate, HDMECs did not express tyrosine hydroxylase, a critical catecholamine synthesis enzyme (Nagatsu et al., 1964) and no adrenaline secretion was detected from HDMECs (results not shown).

**Fig 9:**
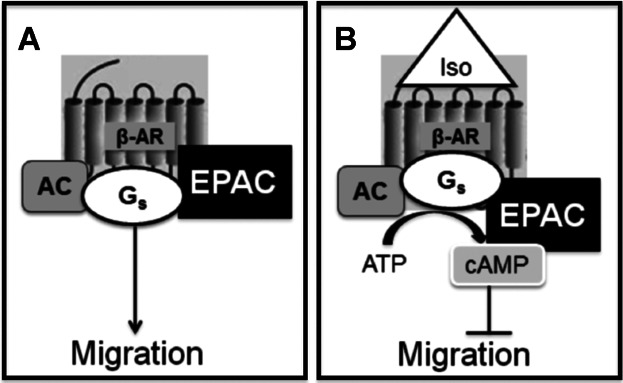
A model to describe the pathways that inhibit HDMEC migration. A β-AR-AC-EPAC complex is present in HDMEC membranes. In the absence of β-AR activation, HDMEC migrate normally (A). Upon β-AR activation, highly localised cAMP is generated which, via EPAC in the complex, inhibits HDMEC migration (B).

Efficient signal transduction downstream of β-ARs, like that of many other GPCRs, involves scaffolding and the subsequent assembly of intracellular signalling complexes at the membrane to bring signalling proteins into proximity, facilitating interactions and rapid signal relay. The β_2_-AR is known to form part of a number of macromolecular signalling complexes; it associates with Gαs, AC, PKA (Hall, 2004) as well as the α-amino-3-hydroxy-5-methyl-4-isoxazolepropionic acid-type glutamate receptor subunit GluR1 (Joiner et al., 2010). In hippocampal neurons, the β_2_-AR associates with Gαs, AC, PKA, protein phosphatase 2 and the Ca^2+^ channel Cav1.2 (Davare et al., 2001) to facilitate the highly localised, rapid, but selective regulation of cAMP signalling pathways. Indeed, Förster resonance energy transfer studies revealed that, upon β-AR activation, cAMP was found in much higher concentrations at the plasma membrane supporting the theory that regulated pools of cAMP become available in a spatial manner upon β-AR activation (DiPilato et al., 2004). In multiple cell types, cAMP can modulate cell migration through the activation of both PKA and EPAC (Gloerich and Bos, 2010); this was replicated in our study. cAMP activation of EPAC leads to its translocation to the plasma membrane where it proximally activates its downstream effector, Rap1 (Ohba et al., 2003; Wang et al., 2006; Ponsioen et al., 2009). Indeed, there is compelling evidence to suggest that the formation of macromolecular signalling compartments directly influence EPAC signalling and cell function (Breckler et al., 2011; Edwards et al., 2012). Therefore, it is reasonable to speculate that a β-AR-AC-EPAC signalling complex could be formed at the HDMEC membrane (Fig. 9A), which, upon β-AR activation, creates a highly localised pool of cAMP capable of reducing HDMEC migration rate through EPAC (Fig. 9B). It was initially surprising that the addition of 8-pCPT did not reduce the HDMEC migration rate alone, but prevented the iso-mediated reduction in motility (Fig. 4C). Perhaps EPAC dissociates from the β-AR complex signalling compartment upon 8-pCPT binding and is, therefore, no longer able to transduce any inhibition of migration in either the absence or presence of β-AR agonist. It is also a possibility that the one-hour incubation with 8-pCPT desensitised or depleted the EPAC signalling system within HDMECs, although after much longer incubations (24 h) EPAC was still active in rat ventricular myocytes (Metrich et al.; 2008). Further studies will investigate this possibility.

Previous research has demonstrated that PKA (Howe, 2004) and EPAC (Lyle et al., 2008; Yokoyama et al., 2008; Grandoch et al., 2009; Baljinnyam et al., 2010) can be both positive or negative regulators of cell migration. EPAC also plays a role in cell polarisation and directional cell migration. In U937 cells, treatment with 8-pCPT-2′-O-Me-cAMP increased the number of polarised cells on fibronectin, spatially distributing EPAC to the rear of the cell and stimulating chemotaxis (Lorenowicz et al., 2006). Indeed, β-AR activation in Ovar3 cells induced integrin-mediated adhesion to fibronectin via a cAMP, EPAC and Rap1-dependent pathway (Rangarajan et al., 2003). However, both PKA and EPAC were required for integrin-mediated adhesion to modulate human umbilical vein endothelial cell migration (Lorenowicz et al., 2008). It appears that cell-type-specific differences exist in the cAMP-dependent pathways used to modulate migration.

Sp cAMP reduced HDMEC single cell migration by 25% (Fig. 4) and decreased angiogenesis in the CAM assay by 51% (Fig. 7). cAMP pathways differ extensively between ECs isolated from different tissues, which could be partly due to differential expression of adenylate cyclase isoforms (Hanoune and Defer, 2001).

In more complex models, β-AR activation reduced angiogenesis. The β-AR agonist-mediated reduction in HDMEC migration and proliferation rate could play a role in the β-AR mediated decrease in tubule formation, embryonic angiogenesis and wound angiogenesis in vivo. In addition, EC recruitment into the wound is mediated by FGF-2 and VEGF, pro-angiogenic growth factors that aid EC migration, proliferation, elongation and alignment (Nissen et al., 1998; Bouis et al., 2006). FGF-2 is synthesised and secreted by a number of wound cells such as dermal fibroblasts and ECs (Schweigerer et al., 1987; Kandel et al., 1991), while keratinocytes are a major source of VEGF in the wound (Brown et al., 1992). β-AR activation decreased FGF-2 secretion from HDMECs and also VEGF-A secretion from HNKs (Fig. 5). It is thus possible that β-AR mediated decreases in pro-angiogenic growth factor secretion from HDMECs and keratinocytes, via autocrine and paracrine mechanisms, contributed to the reduction in angiogenesis observed in vivo.

The amount of wound angiogenesis directly correlates with the level of scarring. Levels of VEGF and angiogenesis are lower in scar-free foetal wounds (Wilgus et al., 2008) and non-scarring oral wounds (Szpaderska et al., 2005), while VEGF addition to wounds promotes scarring (Wilgus et al., 2008). Increased angiogenesis is also associated with pathophysiological scarring. Excessive angiogenesis occurs in hypertrophic scars from 12 weeks post-surgery (van der Veer et al., 2011) and keloids have upregulated vasculature and increased VEGF levels (Mogili et al., 2012). Current treatment options for hypertrophic scars and keloids are very limited and it is possible that the β-AR-mediated reduction in wound angiogenesis in murine wounds, 5-days post-wounding, could contribute to an overall reduction in wound scarring. Unfortunately, the study ended at this early time-point, so it was not possible to determine any alterations to healing and scarring as wound repair progressed. However, in recent work from our lab using a porcine wound model, β-AR activation reduced angiogenesis within 7 days of wounding and by study end, 56 days post-wounding, scar area was reduced by almost 50% (Pullar, Personal communication). Future work will further explore the ability of β-AR activation to reduce angiogenesis and scarring in both normal wounds and pathophysiological scarring, such as hypertrophic scars and keloids.

In contrast, in infantile hemangiomas, vascular tumours that affect 5–10% of all infants, Iso has been shown to increase the proliferation of hemangioma-derived ECs and increased VEGF expression (Ji et al., 2013). Indeed, propranolol, a non-selective β-AR antagonist, is the first-line treatment for infantile hemangiomas (Xiao et al., 2013). Similarly, in oxygen-induced retinopathy, a model for human retinopathy of maturity (Martini et al., 2011), the pathogenic angiogenesis was partly dependent on the β_2_-AR. Indeed, β_2_-AR blockade decreased retinal levels of pro-angiogenic growth factors and decreased pathogenic neovascularisation (Martini et al., 2011). It appears that the β-AR-mediated modulation of angiogenesis is complex and dependent on a number of factors; this study adds another facet to our understanding.

In conclusion, cAMP-dependent mechanisms underpinned the β-AR-mediated anti-motogenicity in HDMECs. A cAMP-dependent but PKA-independent mechanism underpinned the β-AR agonist-mediated reduction in HDMEC motility. β-AR activation also reduced pro-angiogenic growth factor secretion from ECs and keratinocytes. All of these mechanisms could contribute to the β-AR-mediated reduction in HDMEC tubule formation in vitro and chick embryonic and murine skin wound angiogenesis in vivo. The reduction in normal wound angiogenesis mediated by β-AR agonism could have the potential to reduce wound scarring and may thus be useful clinically, particularly in hypertrophic scarring and keloids, known to have upregulated vasculature.

## Financial Disclosure

No funding bodies had any role in study design, data collection and analysis, decision to publish, or preparation of the manuscript. This work was supported by a Wellcome Trust (http://www.wellcome.ac.uk) grant 85586 (CEP), an MRC (http://www.mrc.ac.uk) grant G0901844 (CEP), a BSF (http://www.britishskinfoundation.org.uk) grant 929 s (CEP) and an NIH (NIAMS) grant K01 AR48827 (CEP) (www.niams.nih.gov).

## Author Contributions

CEP initiated the concept for all studies, designed the majority of the studies, interpreted some of the data and drafted, proofed and revised the article. APO designed some of the studies, acquired and analysed the majority of the data and assisted in both the drafting and the proofreading of the article. JMF assisted in the drafting, proofreading and revision of the manuscript.

## Abbreviations

8-CPT-2′-O: Me-cAMP
AC: Adenylate cyclase
β-AR: Beta adrenoceptor
BME: Basement membrane extract
CAM: Chick chorioallantoic membrane
cAMP: Cyclic adenosine monophosphate
EC: Endothelial cell
ECGM: Endothelial cell growth medium
ELISA: Enzyme-linked immunosorbent assay
EPAC: Exchange protein directly activated by cAMP
FGF: Fibroblast growth factor
GPCR: G protein-coupled receptor
HBSS: Hank's balanced salt solution
HDMEC: Human dermal microvascular endothelial cell
HNK: Human neonatal keratinocyte
PBS: Phosphate buffered saline
PKA: Protein kinase A
Rap: Ras-associated protein
SCM: Single cell migration
VEGF: Vascular endothelial growth factor
